# lncRNA-mediated ceRNA network in bladder cancer

**DOI:** 10.1016/j.ncrna.2022.12.002

**Published:** 2022-12-14

**Authors:** Kun Li, Tongyue Yao, Ziqiang Wang

**Affiliations:** a Department of Nuclear Medicine, The First Affiliated Hospital of Shandong First Medical University & Shandong Provincial Qianfoshan Hospital, Jinan, 250014, China; b Biomedical Sciences College & Shandong Medicinal Biotechnology Centre, Shandong First Medical University & Shandong Academy of Medical Sciences, Jinan, 250062, China

**Keywords:** Bladder cancer, lncRNA, miRNA, Competing endogenous RNA, Therapeutic target

## Abstract

Bladder cancer is a common disease associated with high rates of morbidity and mortality. Although immunotherapy approaches such as adoptive T-cell therapy and immune checkpoint blockade have been investigated for the treatment of bladder cancer, their off-target effects and ability to affect only single targets have led to clinical outcomes that are far from satisfactory. Therefore, it is important to identify novel targets that can effectively control tumor growth and metastasis. It is well known that long noncoding RNAs (lncRNAs) are powerful regulators of gene expression. Increasing evidence has shown that dysregulated lncRNAs in bladder cancer are involved in cancer cell proliferation, migration, invasion, apoptosis, and epithelial-mesenchymal transition (EMT). In this review, we focus on the roles and underlying mechanisms of lncRNA-mediated competing endogenous RNA (ceRNA) networks in the regulation of bladder cancer progression. In addition, we discuss the potential of targeting lncRNA-mediated ceRNA networks to overcome cancer treatment resistance and its association with clinicopathological features and outcomes in bladder cancer patients. We hope this review will stimulate research to develop more effective therapeutic approaches for bladder cancer treatment.

## Highlights

• Numerous lncRNAs are dysregulated in bladder cancer.
• These lncRNAs involved in cell proliferation, migration, invasion, apoptosis, cell cycle, EMT, metastasis.
• These lncRNAs act as ceRNA by sponging miRNAs altering expression of their target genes.
• Feedback loop between lncRNAs/miRNA/target can be targeted for bladder cancer therapy.

## 1 Introduction

Bladder cancer is the most common and fatal type of urinary tumor, and its incidence has gradually increased in recent years [1]. Currently, surgical resection, radiotherapy, and chemotherapy are the main treatment strategies for bladder cancer. However, patients undergoing these treatments often experience tumor recurrence and metastasis because of muscle invasion of bladder cancer cells and drug resistance [2]. Although immunotherapy approaches including adoptive T-cell therapy and immune checkpoint blockade have been investigated in bladder cancer because of its frequent mutation, the clinical outcomes are far from satisfactory owing to the off-target effects and limitation to a single target [[3], [4], [5]]. Therefore, there is an urgent need to further understand the molecular mechanisms underlying the progression of bladder cancer and develop a more effective therapeutic approach for this disease.

Long non-coding RNAs (lncRNAs) are non-protein-coding RNA molecules that are greater than 200 nucleotides in length. Accumulating evidence suggests that lncRNAs play an important regulatory role in gene expression at the transcriptional and post-transcriptional levels [[6], [7], [8], [9], [10], [11], [12], [13], [14], [15], [16], [17], [18]]. One of the well-characterized mechanisms of lncRNAs is their action as competing endogenous RNAs (ceRNAs) to “sponge” complementary microRNAs (miRNAs), facilitating the de-repression of their target molecules [19]. In bladder cancer, several oncogenic lncRNAs have been demonstrated to exhibit abnormal expression, which contributes to cancer initiation and progression and is associated with poor prognosis and survival rate by regulating various processes, including proliferation, migration, invasion, epithelial-mesenchymal transition (EMT), cell cycle arrest, and apoptosis [20,21]. There is growing evidence that lncRNAs modulate the expression of tumor-related genes by interacting with or sequestering miRNAs, preventing the degradation or translational inhibition of tumor-related gene transcripts [22]. This suggests that lncRNA-mediated ceRNA networks have exciting potential as therapeutic targets for bladder cancer.

In this review, we discuss the roles of lncRNA-mediated ceRNA networks in bladder cancer progression, with an emphasis on the promotion of tumor cell proliferation, migration, invasion, EMT, cell cycle arrest, apoptosis inhibition, cancer cell stemness, and cytoskeleton rearrangement. We also discuss the potential clinical applications of lncRNA-mediated ceRNA networks in overcoming chemo- and radio-resistance in cancer treatment, as well as their association with patients' clinicopathological features and outcomes.

## 2 The mechanistic function of lncRNAs as ceRNAs in bladder cancer

**Fig. 1:**
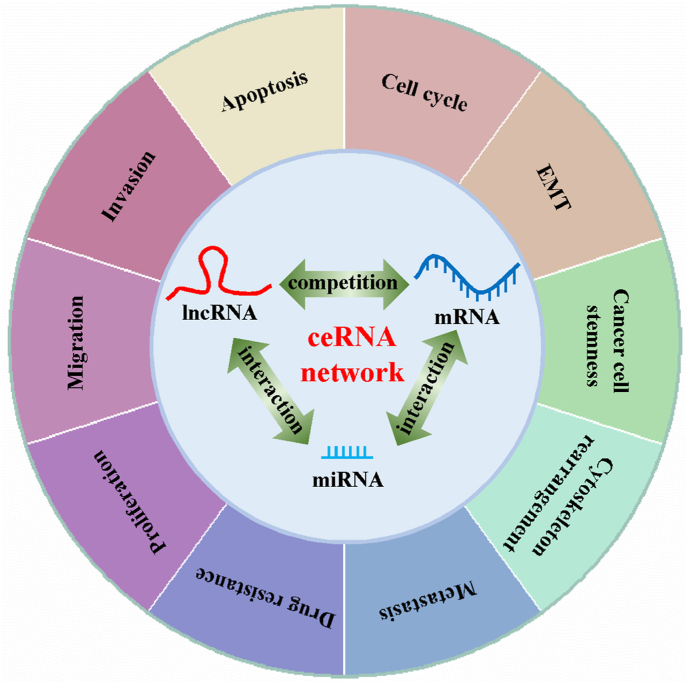
**The mechanistic function of lncRNAs as ceRNAs in bladder cancer.** This schematic model shows that the lncRNA-mediated ceRNA network is important in the regulation of cell proliferation, migration, invasion, apoptosis, cell cycle, EMT, cancer stem cells, and drug resistance in bladder cancer.

An increasing number of studies have demonstrated the dysregulation of the expression of more than 100 lncRNAs in bladder cancer tissues, and some studies have shown that the abnormal expression of lncRNAs is strongly associated with bladder cancer progression [23]. To date, lncRNA-mediated ceRNA networks have been reported to be involved in cell proliferation, migration, invasion, apoptosis, cell cycle, EMT, metastasis, cancer cell stemness, cytoskeleton rearrangement, and drug resistance in bladder cancer (Fig. 1). In the following sections, we summarize and discuss the role of the lncRNA-mediated ceRNA network in regulating these cellular processes.

## 3 lncRNA-mediated ceRNA network in the progression of bladder cancer

To date, numerous lncRNAs have been reported to regulate one or more cellular processes in the progression of bladder cancer, including cell proliferation, invasion, migration, apoptosis, cell cycle, EMT, and tumor metastasis (Table 1). This section describes the functions and mechanisms of several lncRNA-mediated ceRNA networks involved in these cellular processes.

**Table 1:** **Roles of lncRNA-mediated ceRNA network in the progression of bladder cancer**.

LncRNA	Expression	Downstream target	Role	Ref.
UCA1	Up	miR-145/ZEB1/2/FSCN1	Migration↑, invasion↑	[29]
	Up	miR-143/HMGB1	EMT↑	[30]
	Up	miR-195/ARL2	Mitochondrial function↑	[31]
	Up	miR-16/GLS2	Mitochondrial glutaminolysis↑	[33]
	Up	miR-143/HK2	Mitochondrial glucose metabolism↑	[32]
XIST	Up	miR-124/AR	Proliferation↑, invasion↑, migration↑	[35]
	Up	miR-139–5p/Wnt1	Proliferation↑, cells cycle↑, apoptosis↓, metastasis↑	[38]
MALAT1	Up	miR-34a/CCND1	Proliferation↑, migration↑	[41]
	Up	miR-125b/Bcl-2/MMP-13	Invasion↑, apoptosis↓	[40]
PVT1	Up	miR-194–5p/BCLAF1	Proliferation↑, migration↑, apoptosis↓	[45]
	Up	miR-31/CDK1	Proliferation↑, invasion↑, migration↑	[46]
	Up	miR-128/VEGF-C	Proliferation↑, migration↑	[44]
SNHG3	Up	miR-515–5p/GINS2	Proliferation↑, invasion↑, migration↑, EMT↑	[49]
SNHG6	Up	miR-125b/Snail1/2/NUAK1	Invasion↑, migration↑, EMT↑	[51]
SNHG7	Up	miR-2682–5p/ELK1	Proliferation↑, invasion↑, migration↑	[54]
SNHG14	Up	miR-211–3p/ESM1	Proliferation↑, invasion↑, migration↑, cells cycle↑, apoptosis↓	[56]
BCAR4	Up	miR-370–3p/Wnt7a	Proliferation↑, apoptosis↓	[59]
	Up	miR-644a/TLX1	Proliferation↑, invasion↑, migration↑	[60]
CASC9	Up	miR-497–5p/FZD6	Proliferation↑, invasion↑, migration↑, metastasis↑	[64]
	Up	miR-758–3p/TGFβ2	Proliferation↑, invasion↑, migration↑, EMT↑	[66]
DANCR	Up	miR-149/MSI2	EMT↑	[70]
	Up	miR-335/VEGF-C	Proliferation↑, invasion↑, migration↑, cells cycle↑, metastasis↑	[72]
KCNQ1OT1	Up	miR-145–5p/PCBP2	Proliferation↑, invasion↑, migration↑, cells cycle↑, apoptosis↓	[75]
	Up	miR-218–5p/HS3ST3B1	Proliferation↑, apoptosis↓,metastasis↑	[76]
PCAT6	Up	miR-143–3p/PDIA6	Proliferation↑, invasion↑, migration↑	[80]
	Up	miR-513a	Proliferation↑, invasion↑, migration↑	[79]
MAGI2-AS3	Down	miR-15b-5p/CCDC19	Proliferation↓, invasion↓, migration↓,	[85]
	Down	miR-31–5p/TNS1	Migration↓, invasion↓	[84]
PTENP1	/	miR-20a/PDCD4	Viability↓, migration↓	[91]
	Down	miR-17/PTEN	Invasion↓, migration↓, apoptosis↑	[90]
ZEB1-AS1	Up	miR-200b/FSCN1	Proliferation↑, invasion↑, migration↑, apoptosis↓	[96]
	Up	miR-27b	Proliferation↑, apoptosis↓	[95]
SOX2OT	Up	miR-200c/SOX2	Proliferation↑, invasion↑, migration↑, EMT↑, metastasis↑	[100]
OXCT1-AS1	Up	miR-455–5p/JAK1	Proliferation↑, invasion↑	[119]
RNF144A-AS1	Up	miR-455–5p/SOX11	Proliferation↑, invasion↑, migration↑	[120]
lncARSR	Up	miR-129–5p/SOX4	Proliferation↑, invasion↑, migration↑, EMT↑	[121]
ZNRD1-AS1	Up	miR-194/ZEB1	Proliferation↑, invasion↑, migration↑, EMT↑	[122]
VIM-AS1	Up	miR-655/ZEB1	Proliferation↑, invasion↑, migration↑, EMT↑	[123]
TUG1	Up	miR-142/ZEB2	Proliferation↑, apoptosis↓	[124]
miR143HG	Down	miR-1275/AXIN2	Proliferation↓, invasion↓, migration↓, cell cycle↓	[125]
RP11-79H23.3	Down	miR-107/PTEN	Proliferation↓, invasion↓, migration↓, cell cycle↓, apoptosis↑	[110]
MBNL1-AS1	Down	miR-135a/PHLPP2/FOXO1	Proliferation↓, apoptosis↑	[126]
PLAC2	Down	miR-663/TGF-β1	Invasion↓, migration↓	[127]
AC114812.8	Up	miR-371b-5p/FUT4	Proliferation↑, invasion↑, migration↑	[128]
ATB	Up	miR-126/KRAS	Proliferation↑, invasion↑, migration↑	[129]
SPRY4-IT1	Up	miR-101–3p/EZH2	Proliferation↑, invasion↑, migration↑, apoptosis↓	[130]
TINCR	Up	miR-7/mTOR	Proliferation↑, invasion↑, migration↑	[131]
TMPO-AS1	Up	miR-98–5p/EBF1	Proliferation↑, invasion↑, migration↑	[132]
DLX6-AS1	Up	miR-223/HSP90B1	Proliferation↑, invasion↑	[133]
GAS6-AS2	Up	miR-298/CDK9	Proliferation↑, cell cycle↑, EMT↑, metastasis↑	[134]
ARAP1-AS1	Up	miR-4735–3p/NOTCH2	Proliferation↑, invasion↑, migration↑	[135]
PlncRNA-1	Up	miR-136/smad3	Proliferation↑, invasion↑, migration↑	[136]
CALML3-AS1	Up	miR-4316/ZBTB2	Proliferation↑, invasion↑, migration↑, cell cycle↑, apoptosis↓	[137]
MNX1-AS1	Up	miR-218–5p/RAB1A	Proliferation↑, invasion↑, migration↑, EMT↑	[138]
NNT-AS1	Up	miR-1301–3p/PODXL	Proliferation↓, invasion↓, migration↓, EMT↑, apoptosis↑	[139]
HNF1A-AS1	Up	miR-30b-5p/Bcl-2	Proliferation↑, apoptosis↓	[140]
TRPM2-AS	Up	miR-22–3p/GINS2	Proliferation↑, apoptosis↓	[141]
SLCO4A1-AS1	Up	miR-335–5p/OCT4	Proliferation↑, invasion↑, migration↑	[142]
ZNFX1-AS1	Up	miR-193a-3p/SDC1	Proliferation↑, invasion↑, migration↑	[143]
KCNMB2-AS1	Up	miR-3194–3p/SAMD5	Proliferation↑, invasion↑, migration↑	[104]
GAS5	Down	miR-21/PTEN	Proliferation↓, apoptosis↑	[144]
LINC00319	Up	miR-4492/ROMO1	Proliferation↑, apoptosis↓	[116]
LINC00612	Up	miR-590/PHF14	Proliferation↑, invasion↑	[145]
LINC01106	Up	miR-3612/ELK3	Proliferation↑, invasion↑, migration↑	[146]
HCP5	Up	miR-29b-3p/HMGB1	Proliferation↑, invasion↑, migration↑	[147]
STYK1-2	Down	miR-146b-5p/ITGA2	Proliferation↓, invasion↓, migration↓	[148]
H19	Up	miR-29b/DNMT3B	Proliferation↑, invasion↑, migration↑, EMT↑	[109]

### 3.1 UCA1

Urothelial carcinoma-associated 1 (UCA1) is a lncRNA that has been previously found to influence the cell cycle, drug resistance, apoptosis, proliferation, migration, and invasion of bladder cancer cells [[24], [25], [26], [27], [28]]. Recent studies have found that UCA1 promotes bladder cancer cell migration and invasion via the miR-145/zinc finger E-box binding homeobox 1 and 2 (ZEB1/2) and miR-145/fascin actin-bundling protein 1 (FSCN1) pathways [29] and promotes the EMT of bladder cancer cells via the miR-143/high mobility group box 1 (HMGB1) pathway [30]. In addition, studies which aimed to understand the roles of lncRNAs in the metabolism of bladder cancer cells found that UCA1 promotes mitochondrial function in bladder cancer via the miR-195/ADP-ribosylation factor-like 2 (ARL2) signaling pathway [31]. Further investigations revealed that UCA1 enhanced mitochondrial glucose metabolism and glutaminolysis by targeting miR-143 to induce the expression of hexokinase 2 (HK2), the major isozyme contributing to aerobic glycolysis, and by targeting miR-16 to induce the expression of mitochondrial glutaminase 2 (GLS2), which is a vital factor for the conversion of glutamine to glutamate [32,33].

### 3.2 XIST

The X-inactive specific transcript (XIST) is a lncRNA derived from the XIST gene and plays a crucial role in X inactivation [34]. Several studies have reported that XIST expression is upregulated in bladder cancer tissues and functions as an oncogenic gene in cancer development. For example, Xiong et al. [35] found that XIST promoted cell growth, invasion, and migration in bladder cancer by acting as a molecular sponge of miR-124 to enhance the expression of androgen receptor (AR), a vital factor in the progression of bladder cancer [36,37]. Hu et al. [38] found that the XIST/miR-139–5p axis promotes cell proliferation, the cell cycle, and metastasis, and that inhibits cell apoptosis in bladder cancer by activating the Wnt/β-catenin signaling pathway.

### 3.3 MALAT1

Metastasis-associated lung adenocarcinoma transcript 1 (MALAT1) is a lncRNA that is upregulated in various cancers, such as breast, pancreatic, lung, colon, prostate, liver, and bladder [39]. MALAT1 plays a functional role in cancer initiation and progression. A study which aimed to describe the interaction between MALAT1 and miR-125b in bladder cancer cells and their roles in cancer development found that MALAT1 directly targeted miR-125b and antagonized miR-125b expression, resulting in inhibition of apoptosis and promotion of invasion of bladder cancer cells by upregulation of the expression levels of the anti-apoptotic genes B-cell leukemia 2 (Bcl-2) and matrix metallopeptidase 13 (MMP13) [40]. In addition, Liu et al. [41] found that MALAT1 promoted the proliferation and migration of bladder cancer cells by modulating the miR-34a/cyclin D1 (CCND1) axis.

### 3.4 PVT1

Plasmacytoma variant translocation 1 (PVT1) expression was reported to be upregulated in various tumors, including lung carcinomas, osteosarcomas, squamous cell carcinomas, stomach carcinomas, liver carcinomas, colorectal carcinomas, and nasopharyngeal carcinomas [42,43]. One study that investigated the relationship between PVT1 and bladder cancer found that PVT1 levels were significantly higher in bladder cancer tissue and were associated with clinical progression and poor prognosis. Further studies demonstrated that PVT1 enhances the proliferation and migration ability of bladder cancer cells by directly interacting with miR-128, increasing the expression of vascular endothelial growth factor C (VEGF-C), a target of miR-128 that functions as a lymphatic vascular system growth factor [44]. In addition, Chen et al. [45] and Tian et al. [46] reported that PVT1 promotes cell growth, migration, and invasion, and that it inhibits cell apoptosis through the miR-194–5p/B-cell lymphoma-2-associated transcription factor 1 (BCLAF1) and miR-31/cyclin-dependent kinase 1 (CDK1) axis.

### 3.5 SNHGs

Small nucleolar RNA host genes (SNHGs) are a group of lncRNAs that are overexpressed in various cancers and increase the proliferation, invasion, EMT, and metastasis of cancer cells [47,48]. Several SNHGs have been shown to be involved in the progression of bladder cancer. For example, Dai et al. [49] reported that the lncRNA SNHG3 is upregulated in bladder cancer tissues and is associated with poor clinical prognosis. Further investigation showed that SNHG3 significantly enhanced the proliferation, invasion, migration, and EMT of bladder cancer cells *in vitro* and *in vivo* through the regulation of GINS complex subunit 2 (GINS2), a member of the GINS complex involved in DNA replication, by “sponging” miR-515–5p [50]. Wang et al. [51] showed that overexpression of SNHG6 promoted the EMT, migration, and invasion capabilities of bladder cancer cells by targeting tumor suppressive hsa-miR-125b, upregulating the expression of Snail1, Snail2, and NUAK family kinase 1 (NUAK1), which are key inducers of EMT and tumor metastasis [52,53]. Wang et al. [54] found that upregulated SNHG7 expression in bladder cancer tissues and cells is positively correlated with poor prognosis in patients with bladder cancer. Mechanistic studies have shown that SNHG7 enhances cell growth, migration, and invasion by increasing the expression level of ELK1, a transcription factor for genes with oncogenic roles in tumorigenesis [55], by acting as a “sponge” of miR-2682–5p. In addition, ELK1 was identified as a transcription factor for SNHG7, suggesting an ELK1/SNHG7/miR-2682–5p feedback loop. Feng et al. [56] reported that SNHG14 enhances cell cycle progression, colony formation, invasion, migration, and proliferation, and that it inhibits apoptosis of bladder cancer cells by targeting miR-211–3p to upregulate the expression of endothelial cell-specific molecule 1 (ESM1), a soluble dermatan sulfate proteoglycan that participates in cancer progression and metastasis [57].

### 3.6 BCAR4

Breast cancer anti-estrogen receptor 4 (BCAR4) is a lncRNA that has been initially identified as anti-estrogen resistant in breast cancer cells [58]. Subsequent investigations have shown that BCAR4 functions as a promoter for various human malignancies, including bladder cancer. Zhang et al. [59] reported that the expression of lncRNA BCAR4 was elevated in breast cancer tissues and that silencing of BCAR4 inhibited cell proliferation and enhanced cell apoptosis by directly “sponging” miR-370–3p, elevating the expression of Wnt7a, a target of miR-370–3p. Wang et al. [60] showed that high expression of BCAR4 is associated with advanced stage and metastasis of bladder cancer and that BCAR4 significantly promotes cell proliferation, migration, and invasion of bladder cancer by directly binding to miR-644a and upregulating the expression of T cell leukemia homeobox 1 (TLX1), an important transcription factor involved in the progression of many cancers [61,62].

### 3.7 CASC9

In one study, lncRNA cancer susceptibility candidate 9 (CASC9) was originally identified as a promoter for the migration and invasion of esophageal cancer cells [63]. Recently, Zhan et al. [64] found that CASC9 expression was markedly elevated in bladder cancer tissue and that high expression levels of CASC9 were associated with high histological grade, late tumor size-node location-metastasis status (TNM) stage, and poor prognosis. Further investigation revealed that CASC9 enhances tumor bladder cancer cell proliferation, invasion, migration, and metastasis by positively regulating the expression of frizzled receptor 6 (Fzd6), a trigger for cancer progression through the Wnt-signaling pathway [65], by targeting and decreasing the expression level of miR-497–5p. In addition, Zhang et al. [66] revealed that upregulated CASC9 in bladder cancer cell lines and specimens promoted the proliferation, EMT, migration and invasion of bladder cancer through upregulating expression of transforming growth factor β2 (TGFβ2), an inducer for EMT [67], by “sponging” and decreasing the expression level of miR-758–3p.

### 3.8 DANCR

Differentiation-antagonizing non-protein coding RNA (DANCR) was first reported to suppress epithelial cell differentiation, and it was recently identified as a promising diagnostic biomarker and therapeutic target for multiple cancers [68,69]. For instance, Zhan et al. [70] found that DANCR expression was significantly upregulated in bladder cancer and positively correlated with advanced TNM stage and higher histological grade. They also demonstrated that the depletion of DANCR inhibited EMT of bladder cancer cells by “sponging” miR-149 and subsequently elevating the expression level of musashi RNA-binding protein 2 (MSI2), a promoter for cancer initiation, progression, and drug resistance [71]. In addition to EMT, another study reported that DANCR significantly promotes the proliferation, invasion, migration, and lymphatic metastasis of bladder cancer cells by targeting miR-335, upregulating the expression of VEGF-C, a target of miR-335 [72].

### 3.9 KCNQ1OT1

KCNQ1 opposite strand/antisense transcript 1 (KCNQ1OT1) is a novel lncRNA that plays a vital role in cancer progression [73]. A study investigating the function of lncRNA KCNQ1OT1 in bladder cancer found that KCNQ1OT1 was significantly overexpressed in bladder cancer tissues and cell lines and that knockdown of KCNQ1OT1 resulted in repressed proliferation, migration, invasion, and enhanced apoptosis of bladder cancer cells. Mechanistic study demonstrated that KCNQ1OT1 “sponged” miR-145–5p and upregulated the expression of poly (rC)-binding protein 2 (PCBP2), a target of miR-145–5p that functions as an oncogene [74,75]. Li et al. [76] reported that KCNQ1OT1 modulated the malignant phenotypes of bladder cancer cells by specifically binding to miR-218–5p and increasing the expression level of heparan sulfate-glucosamine 3-sulfotransferase 3B1 (HS3ST3B1).

### 3.10 PCAT6

Prostate cancer-associated transcript 6 (PCAT6) is a lncRNA that was first identified to participate in the pathoetiology of prostate cancer [77,78]. Recently, PCAT6 expression was found to be upregulated in bladder cancer tissues and cell lines, and high expression of PCAT6 was associated with reduced overall survival. Further mechanistic studies have shown that PCAT6 enhances the viability, migration, and invasion of BC cells by directly interacting with miR-513a [79]. In addition, Zhang et al. [80] demonstrated that the knockdown of PCAT6 resulted in the inhibition of the proliferation, migration, and invasion of bladder cancer cell lines T24T and EJ by targeting the miR-143–3p/protein disulfide isomerase family 6 (PDIA6) axis, where PDIA6 was previously reported to be involved in the pathogenesis of human cancers and the sensitivity of cancer cells to chemotherapeutic drugs [81,82].

### 3.11 MAGI2-AS3

To identify the ceRNAs that regulate bladder cancer progression, a bioinformatic study revealed that MAGI2 antisense RNA 3 (MAGI2-AS3), a dysregulated lncRNA in multiple cancers [83], showed a significantly lower expression in bladder cancer tissues and cell lines and a strong positive correlation with Tensin 1 (TNS1) mRNA expression by targeting miR-31–5p. Further functional experiments demonstrated that the MAGI2-AS3/miR-31–5p/TNS1 axis was correlated with advanced tumor stages and lymph node metastasis through the regulation of the migration and invasion of bladder cancer cells [84]. In addition, Wang et al. [85] found that downregulation of MAGI2-AS3 in bladder cancer tissues promoted proliferation, migration, and invasion of bladder cancer cells by targeting miR-15b-5p and downregulating the expression of CCDC19, which is a potential tumor suppressor in nasopharyngeal carcinoma, non-small cell lung cancers, and lung squamous cell carcinoma [[86], [87], [88]].

### 3.12 PTENP1

Phosphatase and tensin homolog pseudogene 1 (PTENP1), a pseudogene of the tumor suppressor gene PTEN, was the first pseudogene to be characterized as a ceRNA [89]. In a study that aimed to investigate the role of exosomal lncRNA PTENP1 in bladder cancer, exosomal PTENP1 was found to mediate communication between cells in the tumor microenvironment by transferring PTENP1 from normal cells to bladder cancer cells. This increases apoptosis and inhibits the invasion and migration of cancer cells by upregulating PTEN expression in bladder cancer cells by competitively binding to miR-17 [90]. In addition, Zhong et al. [91] reported that lncRNA PTENP1 “sponged” miR-20a to elevate the expression level of programmed cell death 4 (PDCD4), a tumor suppressor that induces apoptosis and inhibits cell growth, invasion, and metastasis [92], resulting in inhibition of the viability and migration of bladder cancer cells.

### 3.13 ZEB1-AS

Zinc finger E-box binding homeobox 1 antisense 1 (ZEB1-AS1) is a novel tumor-associated lncRNA that functions as an oncogenic regulator in many types of cancer [93]. In a study exploring the role of lncRNA ZEB2-AS1 in bladder cancer, the expression of ZEB2-AS1 was found to be significantly increased in bladder cancer tissues and cell lines. Functional investigation showed that upregulated ZEB2-AS1 significantly enhanced the proliferation and inhibited the apoptosis of bladder cancer cells by “sponging” miR-27b, a tumor-suppressive miRNA, during bladder cancer progression [94,95]. Moreover, Gao et al. [96] found that high expression of ZEB1-AS1 in bladder cancer tissues was positively correlated with high tumor grade and TNM stage. Mechanistic studies revealed that ZEB1-AS1 functions as a “sponge” for miR-200b to regulate the expression of FSCN1, a globular actin-bundling protein that is important in cancer cell migration and invasion during tumor progression [97].

## 4 lncRNA-mediated ceRNA network in bladder cancer cell stemness

Emerging evidence has shown that cancer cell stemness, the stem cell-like phenotype of cancer cells, is important in tumorigenesis, metastasis, recurrence, and drug resistance [98,99]. lncRNA-mediated ceRNA networks have been confirmed to participate in the formation and maintenance of cancer cell stemness in human cancers, including bladder cancer. Zhan et al. [100] found that the sex-determining region Y-box2 (SOX2) overlapping transcript (SOX2OT) was highly expressed in bladder cancer and that increased SOX2OT expression promoted self-renewal, migration, invasion, and tumorigenicity of bladder cancer stem cells via “sponging” of miR-200c and subsequent enhancement of the expression of SOX2, a vital regulator of cancer stemness [101,102]. Moreover, the oncogenic lncRNA HOXA cluster antisense RNA 2 (HOXA-AS2) was found to promote the stemness of bladder cancer cells by elevating the expression levels of the cancer stem cell markers ALDH1A1, CD44, HMGA2, KLF4, and OCT4 by regulating the miR-125b/smad2 axis [103]. In addition, the potassium calcium-activated channel subfamily M regulatory beta subunit 2 (KCNMB2) antisense RNA 1 (KCNMB2-AS1) was reported to enhance the stemness of bladder cancer cells by elevating the expression levels of cancer stem cell markers CD133, Nanog, Oct4, Sox2, and ALDH1 via regulation of the miR-3194–3p/smad5 signal pathway [104].

## 5 lncRNA-mediated ceRNA network in cytoskeleton rearrangement

The eukaryotic cytoskeleton is a complex fibrous reticular structure consisting of microfilaments, microtubules, and intermediate filaments. Increasing evidence has demonstrated that the cytoskeleton is responsible for cell division, intercellular transport, motility, and signal transduction, resulting in its role in uncontrolled cell proliferation and migration during cancer progression [105,106]. In bladder cancer progression, the lncRNA-mediated ceRNA network has been reported to rearrange the cytoskeleton. For instance, Lv et al. [107] found that lncRNA H19 is highly expressed in bladder cancer tissues and cell lines and that the overexpression of lncRNA H19 results in rearrangement of the cytoskeleton by upregulating the expression of paxillin and F-actin, two cytoskeletal proteins with crucial roles in signal transduction, motor activity, adhesion, and migration in cancer cells [108]. Further investigation revealed that H19 directly bound to miR-29b and upregulated the expression of DNA methyltransferase 3 B (DNMT3B), a target of miR-29b, which is associated with the initiation and progression of bladder cancer [109]. Moreover, Chi et al. [110] found that lncRNA RP11-79H23.3 is another regulator of cytoskeletal rearrangement through the regulation of paxillin and F-actin. They reported that lncRNA RP11-79H23.3 is downregulated in bladder cancer tissues and cell lines. This downregulation promotes the rearrangement of the cytoskeleton and enhances cell proliferation, migration, and cell cycle progression. Mechanistically, lncRNA RP11-79H23.3 is revealed to upregulate the expression of the tumor suppressor PTEN by directly binding to miR-107. These studies suggest that the lncRNA-mediated ceRNA network could be targeted during bladder cancer therapy by regulating cytoskeletal rearrangements.

## 6 lncRNA-mediated ceRNA network in therapeutic resistance

Conventional treatments for bladder cancer include surgery, chemotherapy, and radiotherapy. However, a subset of patients with bladder cancer are unresponsive to chemotherapy or radiotherapy and consequently experience tumor recurrence [2,111]. Overcoming chemo- and radio-resistance is a major challenge in achieving better outcomes in bladder cancer patients, and several studies have found that lncRNAs are associated with development of resistance to chemotherapy or radiotherapy in bladder cancer through the ceRNA network (Table 2). They found that targeting lncRNA-mediated ceRNA networks could sensitize cancer cells to cisplatin, gemcitabine, and doxorubicin. In addition, TUG1, a lncRNA that is overexpressed in bladder cancer tissues and cell lines, promotes EMT and decreases cancer cell sensitivity to ionizing radiation through the miR-145/ZEB2 axis [112]. Another study revealed TUG1 silencing increased radiosensitivity in a xenograft model by inhibiting expression of HMGB1, a conserved nuclear protein that promotes metastasis in various cancers [113,114]. Moreover, a recent study investigating lncRNA signatures in patients with bladder cancer treated with radiotherapy found that a 10-lncRNA signature is associated with molecular processes involved in radiation responses; knockdown of one of these lncRNAs exhibited a modest increase in radiosensitivity in bladder cancer cells [115]. These studies suggest that targeting the lncRNA/miRNA/target axis is a potential strategy in overcoming therapeutic resistance in bladder cancer.

**Table 2:** **Roles of lncRNA-mediated ceRNA network in the resistance to chemo- and radio-therapy in bladder cancer**.

LncRNA	Expression	Downstream target	Chemical-/radio- resistance	Ref.
TUG1	Up	miR-194–5p/CCND2	Cisplatin	[149]
UCA1	Up	CREB/miR-196a-5p/p27Kip1		[150]
DLEU1	Up	miR-99b/HS3ST3B1		[151]
MST1P2	Up	miR-133b/SIRT1/p53		[152]
MALAT1	Up	miR-101–3p/VEGF-C		[153]
UCA1	Up	CREB/miR-196a-5p/p27Kip1	Gemcitabine	[150]
FOXD2-AS1	Up	miR-143/ABCC3		[154]
LET	Up	NF90/miR-145/HMGA2 and KLF4		[155]
NEAT1	Up	miR-214–3p/Wnt/β-catenin	Doxorubicin	[156]
TUG1	Up	miR-145/ZEB2	Radiation	[112]

## 7 lncRNA-mediated ceRNA network in patients’ clinical-pathological features and outcomes

In addition to understanding the roles and molecular mechanisms of lncRNA-mediated ceRNA networks in regulating the cellular processes of bladder cancer, researchers have evaluated the association of lncRNA-mediated ceRNA networks with clinicopathological features and prognostic value. As shown in Table 3, dozens of ceRNA networks are reported to be associated with tumor size, grade, TNM stage, prognosis, and survival rate in patients with bladder cancer. Most of the related lncRNA expressions are upregulated in bladder cancer tissues and are positively correlated with large tumor size, high histological grade, advanced TNM stage, poor prognosis, and reduced survival rate. Promotion of cell proliferation, invasion, migration, and EMT via related ceRNA networks was reported, while expressions of lncRNA MAGI2-AS3, LINC00319, LINC00641, HAND2-AS1, and miR-497-HG were decreased and exhibited a negative correlation with bladder cancer patients' high histological grade, advanced TNM stage, poor prognosis, and short survival rate via the miR-31–5p/TNS1 axis [84], miR-15b-5p/CCDC19 axis [85], miR-4492/ROMO1 axis [116], miR-197–3p/KLF10 axis [117], miR-146/RARB axis [118], miR-324–5p/regulator of calcineurin 1 [157], and miR-4738–3p/FosB proto-oncogene, AP-1 transcription factor subunit [157].

**Table 3:** **LncRNA-mediated ceRNA network is associated with patient's clinic-pathological features and outcomes**.

LncRNA mediated ceRNA network	Tumor size	Grade	TNM	Prognosis	Survival rate	Ref.
XIST/miR-124/AR			✓			[35]
XIST/miR-139–5p/Wnt1	✓	✓	✓	✓		[38]
MALAT1/miR-34a/CCND1			✓			[41]
BCAR4/miR-644a/TLX1			✓			[60]
CASC9/miR-497–5p/FZD6		✓	✓	✓		[64]
CASC9/miR-758–3p/TGFβ2				✓		[66]
DANCR/miR-149/MSI2		✓	✓			[70]
PCAT6/miR-513a					✓	[79]
MAGI2-AS3/miR-15b-5p/CCDC19				✓		[85]
MAGI2-AS3/miR-31–5p/TNS1			✓			[54]
LINC00319/miR-4492/ROMO1				✓		[116]
LINC00641/miR-197–3p/KLF10				✓		[117]
SNHG3/miR-515–5p/GINS2				✓		[50]
SNHG7/miR-2682–5p/ELK1				✓		[55]
HAND2-AS1/miR-146/RARB		✓				[118]
ZEB1-AS1/miR-200b/FSCN1		✓	✓		✓	[97]
SOX2OT/miR-200c/SOX2		✓	✓	✓		[100]
RNF144A-AS1/miR-455–5p/SOX11				✓		[120]
lncARSR/miR-129–5p/SOX4	✓	✓				[121]
miR143HG/miR-1275/AXIN2					✓	[125]
PLAC2/miR-663/TGF-β1					✓	[127]
TINCR/miR-7/mTOR			✓		✓	[131]
TMPO-AS1/miR-98–5p/EBF1				✓		[132]
GAS6-AS2/miR-298/CDK9			✓	✓		[134]
ARAP1-AS1/miR-4735–3p/NOTCH2					✓	[135]
PlncRNA-1/miR-136/smad3			✓			[136]
CALML3-AS1/miR-4316/ZBTB2			✓	✓		[137]
SLCO4A1-AS1/miR-335–5p/OCT4			✓	✓		[138]
ZNFX1-AS1/miR-193a-3p/SDC1	✓		✓			[143]
GAS5/MiR-21/PTEN					✓	[144]

## 8 Conclusion and future perspectives

**Fig. 2:**
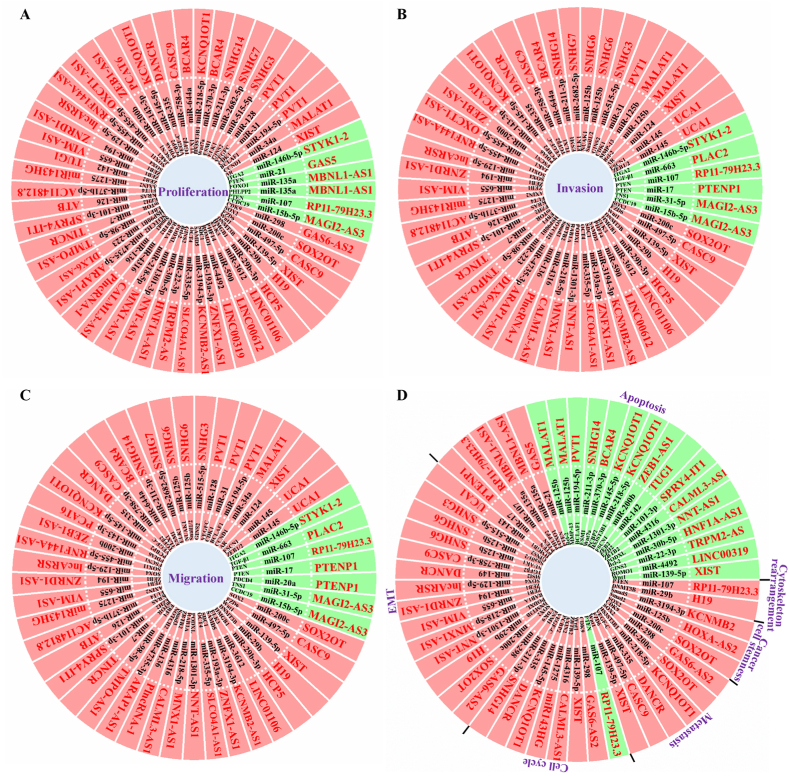
**The lncRNA-mediated ceRNA network contributes to the regulation of bladder cancer progression.** Numerous lncRNA-mediated ceRNA networks have been reported to regulate tumor cells proliferation (A), invasion (B), migration (C), EMT (D), cell cycle (D), apoptosis (D), metastasis, cancer cell stemness (D), and cytoskeleton rearrangement (D) in bladder cancer. Red characters indicate lncRNAs. The pink part indicates the lncRNA-mediated ceRNA network that promotes these cellular processes, and the green part indicates the lncRNA-mediated ceRNA network with an inhibitory role.

Globally, bladder cancer is the leading cause of death from malignancy. Over the past few years, several lncRNAs have been reported to be aberrantly expressed in bladder cancer, most of which promote tumorigenesis and cancer progression. This review summarized the existing information regarding the roles of lncRNA-mediated ceRNA networks in the progression of bladder cancer (Fig. 2). Targeting of lncRNA-mediated ceRNA networks has revealed their therapeutic potential in increasing the efficacy of radiotherapy and chemotherapy. Therefore, lncRNA-mediated ceRNA networks are of relevant research interest, and their role in bladder cancer therapies should be considered. However, as most bladder cancer-related lncRNAs are located in the nucleus, researchers should aim to clarify how these lncRNAs “sponge” miRNAs to regulate their downstream target genes. In addition, more studies are needed to characterize the roles of lncRNA-miRNA networks in tumor microenvironments, such as whether they affect the function of tumor-infiltrating lymphocytes. More importantly, much attention should be directed toward synthesizing a “lncRNA” to sequester oncogenic miRNAs as a therapeutic approach for bladder cancer treatment.

Numerous reports have documented that lncRNAs are a type of less conserved molecule [158,159] and exert their functions in transcriptional and post-transcriptional gene regulation by forming specific structures and interacting with proteins and nucleic acids, indicating that lncRNA structures are vital to their functions [[160], [161], [162]]. In lncRNA-mediated ceRNA networks, lncRNA and mRNA co-regulate each other by competitively binding to shared miRNAs depending on their secondary structure [[163], [164], [165]]. Therefore, to fully understand the impact of lncRNA-mediated ceRNA networks on the development of bladder cancer and design appropriate therapeutic intervention based on ceRNA crosstalk, it is important to resolve lncRNA structures and characterize the interactions of lncRNAs with molecular partners while experimental and computational technology for RNA structure identification is developed. We hope this insight into the roles of lncRNA-mediated ceRNA networks in the progression of bladder cancer will assist in leading to their potential clinical utility in cancer treatment.

## Author contributions

Ziqiang Wang, Kun Li, and Tongyue Yao: Conceptualization, Writing - original draft. Ziqiang Wang: Supervision, Writing - review & editing.

## Declaration of competing interest

The authors declare no conflict of interest.

## 9. Abbreviations

lncRNAs: long noncoding RNAs
EMT: epithelial-mesenchymal transition
ceRNA: competing endogenous RNA
miRNAs: microRNAs
UCA1: urothelial carcinoma-associated 1
ZEB1/2: zinc finger E-box binding homeobox 1 and 2
FSCN1: fascin actin-bundling protein 1
HMGB1: high mobility group box 1
ARL2: ADP-ribosylation factor-like 2
HK2: hexokinase 2
GLS2: glutaminase 2
XIST: X-inactive specific transcript
AR: androgen receptor
MALAT1: metastasis-associated lung adenocarcinoma transcript 1
Bcl-2: B-cell leukemia 2
MMP13: matrix metallopeptidase 13
CCND1: cyclin D1
PVT1: plasmacytoma variant translocation 1
VEGF-C: vascular endothelial growth factor C
CDK1: cyclin-dependent kinase 1
BCLAF1: b-cell lymphoma-2-associated transcription factor 1
SNHGs: Small nucleolar RNA host genes
GINS2: GINS complex subunit 2
NUAK1: NUAK family kinase 1
ESM1: endothelial cell-specific molecule 1
BCAR4: breast cancer anti-estrogen receptor 4
TLX1: T cell leukemia homeobox 1
CASC9: cancer susceptibility candidate 9
Fzd6: frizzled receptor 6
TGFβ2: transforming growth factor β2
DANCR: differentiation-antagonizing non-protein coding RNA
MSI2: musashi RNA-binding protein 2
KCNQ1OT1: KCNQ1 opposite strand/antisense transcript 1
PCBP2: poly(rC)-binding protein 2
HS3ST3B1: heparan sulfate-glucosamine 3-sulfotransferase 3B1
PCAT6: prostate cancer-associated transcript 6
PDIA6: protein disulfide isomerase family 6
MAGI2-AS3: MAGI2 antisense RNA 3
TNS1: Tensin 1
PTENP1: phosphatase and tensin homolog pseudogene 1
PDCD4: programmed cell death 4
ZEB1-AS1: zinc finger E-box binding homeobox 1 antisense 1
SOX2: sex-determining region Y-box2
SOX2OT: SOX2 overlapping transcript
HOXA-AS2: HOXA cluster antisense RNA 2
KCNMB2: potassium calcium-activated channel subfamily M regulatory beta subunit 2
KCNMB2-AS1: KCNMB2 antisense RNA 1
DNMT3B: DNA methyltransferase 3 B
TNM: Tumor size-Node location-Metastasis status
